# Thermometry in Health and Disease

**Published:** 1873-01

**Authors:** F. C. Curtis

**Affiliations:** Albany, New York


*Thermometry in Health and Disease.* By F. C. CURTIS, M.D., Albany, New York.

Within a few years, comparatively, various mechanical contrivances have been devised for assisting in the diagnosis and prognosis of diseases, and for obtaining indications for treatment. The thermometer is one of these physical assistants, the clinical use of which is the subject of this paper. Like all the others, it is only an assistant, but is at least that, and I believe its use should be more extensive than it is. Outside of the hospitals I doubt if it is used much, and, while the hospital is the place to study the instrument, because the patients are there in a body and always close at hand, still it is very portable; and, although regularity of observation is a vital point in its use, yet a single observation is often of great value.

The instrument used is a modification of the ordinary mercurial thermometer. A thermo-electric apparatus has been devised which surpasses in delicacy the best mercurial instrument, and by which the temperature of internal parts can be obtained. It consists of two wires, of different metals, soldered together, and having their free ends brought into communication with a thermo-electric multiplier. The wires are passed into the tissues like acupuncture-needles, and the temperature of the point of contact of the two metals is indicated. It has thus been found that the body is cooler toward the surface. But for obvious reasons this apparatus is only adapted to scientific investigation, and the mercurial thermometer is the only instrument of practical usefulness.

Great care should be used in getting an accurate thermometer, especially if the observations are to be preserved for reference. There may be good American instruments made, but I have never seen one. I found in the New York Hospital three, of New York makers, all of which varied nearly a degree. A fault which renders an instrument nearly useless, is an unequal variation between different degrees. I believe the best—most accurate as well as portable—is that made by Casella, of London. The Germans make very good instruments, but as a rule they are graduated to the scale of Reaumur, or Centigrade. Casella's are carefully made and tested, and are graduated to fifths of a degree. The instrument should be sufficiently delicate to reach its maximum in three minutes, except in a very emaciated subject; some will continue to rise for ten minutes. It is best to have it self-registering. In these, the index consists of half an inch of mercury detached from the rest by a minute speck of air. This avoids the necessity of stooping over in close contact with the patient in such diseases as typhoid, which are more or less contagious; the sick-room, too, is often darkened so that it is not easy to read it *in situ*; it can be trusted to the nurse for one of the daily observations better than the non-registering instruments.

The axilla is the usual point for taking the temperature, and if another point is chosen, it should be noted, as the temperature differs. It should be properly inserted. On the brachial side of the axilla, a little down, it will be noticed that a fold forms as the arm is carried across the body. Lay the bulb in this place, first drawing the clothing well away, and then carry the hand over to the opposite shoulder, the object being to surround the bulb entirely by living tissue. With female patients who may feel a delicacy toward exposure, direct the nurse to lay a towel over the chest before opening the clothing enough to insert the bulb.

Although single observations are often valuable, it is much better to take them continuously. The relation of morning to evening temperature, and the range from day to day, are very valuable, the main usefulness of the instrument being here. If taken continuously they should be regularly, from 7 to 9 o'clock in the morning, and 5 to 7 o'clock in the evening.

The clinical thermometer indicates pyrexia and measures its amount with accuracy. The value of thus accurately determining the amount of heat in disease was first insisted on by De Haen, of Vienna, about a hundred years ago. Heat of body above the normal has been noted as indicating disease ever since medicine was practiced, and is generally recognized by the people as pathological. But the only indication of it has been the hand of the doctor, or the sensations of the patient. Neither of these is reliable; the former is only comparative, varying with the warmth of the hand—the latter cannot be depended on, as is shown in a chill, when the body feels cold to the patient, though really the temperature is elevated. I had been taking the temperature of a patient with erysipelas for several days, the case had gone on to convalescence, when, one evening as I came to take it again, I found the patient in a chill initiatory of relapse, and while in it I found the temperature above what it had been, and 2° above normal. It may be that in these cases the skin is really colder from the nervous contraction of the blood-vessels, but the blood is warmer, and begins to be so before the chill comes on.

But, aside from the mere detection of pyrexia, which of course can in most cases be detected without an instrument, there is the accuracy of measuring it which can be gained by it alone. The value of this I can show farther on, in getting the typical range of diseases, in finding the extremely high ranges, as 108° or 110°, which by the hand cannot be distinguished from considerably lower ones, etc. Anything which makes medicine less an art and more a science, and does away, in any degree, with the vast amount of uncertainty which surrounds our practice, ought to be cherished as it deserves.

Pyrexia is abnormal elevation of temperature. This definition implies the possibility of changes within the bounds of health, and such is the case. The normal temperature of the axilla of adults is 98.5°, and the range of healthy fluctuation is quite limited. Climate, exercise, different hours of the day, are some of the agents affecting it. Dr. John Davy, who experimented largely, found variations between temperate and tropical regions. The average temperature is nearly a degree higher in tropical climates. In temperate, the *maximum* temperature is found in the early morning, being on the average 98.7°; falling steadily through the day until midnight, when it is 97.9°. In tropical regions the minimum temperature is in the morning, being 98.05° on an average, from which it rises steadily until night to 99°, a variation of nearly a degree. The variations of temperature of the air, do not necessarily affect it, though exposure to extreme heat or cold may do so. Active exercise short of fatigue raises it; beyond this point it lowers it. It falls after a full meal, but rises as digestion advances. It is thus seen that variations of temperature between 98° and 99° do not necessarily indicate disease.

There are other variations, peculiar to early life, to the puerperal state, which is in the border-line between physiology and pathology, and also there is difference of temperature in different parts of the body. Dr. Forster gives, in the *Journal fur Kinderkrankheiten*, 1862, the result of an extensive series of observations on the newly born, the temperature being taken in the axilla. At the moment of birth the temperature is the same as that of the mother; it falls directly until in from fifteen minutes to two hours it reaches 28.97° Reaumur or 97.18° Fahr., the average of all taken; the lowest taken was 95.5°. After this fall, immediately following birth, the temperature gradually rises for thirty-six hours, reaching a height of 99.6° Fahr. After the thirty-sixth hour it falls somewhat, but slowly, for four days, when it reaches about the temperature of the healthy adult. It soon rises again, from the fifth to the eighth day, but this oscillation is slight in amount, reaching an average of 99°. It falls somewhat after this, but the temperature of young children is, as a rule, higher than that of adults. The daily range too is greater, amounting to a variation, according to Mr. Finlayson, of 2°. According to the same observer, there is invariably an evening fall in temperature, being lowest between 7 and 9 P. M., and highest about 4 A. M., there being during the day but trifling variation. These normal variations should be borne in mind when using the thermometer in children.

In the later months of pregnancy the temperature of the body is somewhat increased, according to Mr. Squier, in the London *Lancet*. It is not raised much by a natural labor, any more than strong exertion of any other kind affects the person in health. Toward the close of labor the vagina shows an elevated temperature, in one case being noted as high as 99.9°. In prolonged labor, where muscular exertion is severe and continued, a temperature of 102° is sometimes reached, which can hardly be called normal. It subsides soon after labor. The slight febrile action coming on two or three days after labor, called milk-fever, has an elevation of temperature variable both as to height and duration. It is more prolonged in primiparæ, and has been noted as high as 102° or even 104.3°, lasting in the latter case for twelve days, though the breasts were not affected. A sore nipple, or slight inflammation of the breast, raises the temperature.

Different parts of the body have different temperatures. There is a difference of 1° between the covered parts of the surface, as the axilla, where the bulb may be entirely surrounded by living tissue, and the cavities which open externally—the mouth, rectum, and vagina. I believe the lungs are cooler than the abdominal organs, because of the entrance of outside air, and there is a difference in temperature of the blood in the two sides of the heart. The blood coming to the heart by the superior vena cava is of lower temperature than that of the inferior. A recent number of the London *Lancet* contains the interesting fact, that the cranial cavity is of lower temperature than the body generally. There is a difference of $\frac{7}{10}$° Centigrade, or more than one degree Fahr., between the cranial temperature and that of the rectum. Chloroform lowers the temperature of the body generally, and this is more marked in the cranium. The same is true of chloral, and of morphia in large doses, though small doses increase it. But in poisoning by alcohol, the cranial temperature rises above that of the rectum. The experiments were made on dogs and other lower animals, but how the temperature of the cranium was taken is not stated. Mr. Blake, (*Medical Times and Gazette*,) found a difference of from $\frac{1}{2}$° to 1° in the left side of the body over the right, but only during exertion. It was more marked when exercising under a powerful sun.

In this connection I may also speak of *post-mortem* elevation of temperature. De Haen, who first used the thermometer, observed a rise of temperature in the death-agony, which persisted after death. The point being forgotten, was recently re-established, and a prize-article written on it by Valentin, at the University of Berne. Experiments were made on various lower animals. It was found to be true in all cases. It is due both to an increased development of heat, and to diminished loss of heat. The vital processes which ordinarily develop heat during life persist after death—that is, after the heart ceases to beat—especially certain nervous influences ; and probably *post-mortem* disintegration and decomposition may aid in the development of heat. *Rigor mortis* is thought to have but little influence. It would be interesting to know how the *post-mortem* temperature is after sunstroke, in which disease the body-heat is very high, and after death there is no *rigor mortis.*

The retention of heat after death is favored by the cessation of perspiration, and the consequent lessened evaporation from the skin. Evaporation of perspiration is the safety-valve in life to those exposed to heat. As long as this is active, there is little danger of the high ranges of temperature. In sunstroke it is stopped, and when the body-heat reaches 110°, as reported by Wilson Fox in rheumatism, the perspiration, which is usually excessive in this disease, has stopped.

I have thus, somewhat rapidly, sketched those variations and conditions of thermometry which are outside of that portion of the subject which considers them in the sick patient. We find the healthy body maintaining a temperature varying very little, though surrounded by an atmosphere which changes from day to day, which in the course of the year goes through wide ranges, and in the torrid zone has a constant temperature very far removed from that of the frigid. The source of this animal heat is in the tissues themselves, in which there is constantly going on an endless variety of solutions, combinations, and decompositions, by means of which is effected the metamorphosis of food into tissue, and of tissue into effete matter. But the amount of this heat-producing tissue-change varies continually; to prevent a corresponding increase of heat, we have the cutaneous perspiration, the evaporation of which causes the heat to become latent. So the temperature is continually regulated, and the almost unavarying standard maintained. Variation from the standard in disease is due to change in the one or the other, or of their relation to each other.

What is the advantage afforded by the use of the thermometer in disease?

In the first place, it shows whether or not pyrexia actually exists. This seems to be a small matter, but really it is often a valuable item of knowledge. Often diagnosis is very difficult, especially with children, who may present grave symptoms on slight causes. For instance, not long ago I saw a boy ten years of age who had been taken with convulsions, headache, vomiting, and loss of appetite; the bowels were regular, the pulse irritable, but not accelerated, and he lay quietly in bed, with a peculiar nervous look of the eyes. I found his temperature quite normal, and concluded that his convulsions were due to peripheral irritation, not to central lesion. His convulsions were relieved by a full cathartic, and their cause was found to be eating hard Bologna sausage. There are many cases where the thermometer will give this negative help.

It has another advantage which belongs to it alone; it shows, *before other symptoms can*, the existence of disease and changes in the course of disease. The temperature of the body is more sensitive to diseased action than either the pulse or respiration. In intermittent fever the temperature begins to rise several hours before the exacerbation comes on, and after the disease has been reduced by treatment, and all other symptoms have disappeared, a periodic rise of temperature may be noted, showing that the cure is not yet perfect. In typhoid fever the daily rise and fall of temperature is very regular, and any marked deviation from this is always premonitory of something new in the course of the disease. A sudden reduction is said to show that hæmorrhage of the bowels has taken place, from sloughing of Peyer's patches, and several days may elapse before we get evidence of it from the stools. In pneumonia a sudden fall in temperature about the seventh day shows that convalescence has set in, though other symptoms may be as grave as ever. In all acute diseases a fall in temperature is, as a rule, a favorable sign.

The converse is in like manner true; rise of temperature, after it has once fallen, points to a relapse. We see this mainly in diseases, as erysipelas, which are liable to relapses. This rise will be found to take place before the premonitory chill, and will be a marked elevation above the regular rise and fall of the steady course of the disease.

There are two or three general rules of temperature in disease which may be spoken of here: First, there is, as a rule, evening exacerbation, the evening temperature being higher than the morning. In some acute diseases, however, the line of thermometric range is steadily upward for a few days, as if carried along by the impulse of the disease. But usually, even in the most acute diseases, there is a fall in the morning from the temperature of the preceding evening; we know that it is common for all sick people to feel better in the morning, and worse as the afternoon wears away.

There is another peculiar fact, familiar to all who use the instrument, though I have not seen it noted in the books. In some acute diseases the temperature falls *below* the normal standard of health as soon as the acute stage is passed. It has been noted below 95° under these circumstances. There are but few other conditions in which a temperature below that of health is noted. Dr. E. R. Hun found it so low as 94.5° in a case of Bright's disease, in which the œdema was excessive, a few hours before convulsions occurred, in which the patient shortly afterward died. The lowest temperatures have been found in shock. This, from all causes, operations, fractures, burns, etc., seems to be attended with more or less depression of temperature. In the "Biennial Restrospect" of the New Sydenham Society, for 1871, Mr. Wagstaffe is recorded as having found, in a case of cut-throat which recovered, a depression to 91.2°, due probably to loss of blood. In a fatal case of fractured spine, a fall of 16.65° was noted. This gives us a hint that nervous influences have a part in maintaining and regulating animal heat.

The thermometer is a help in prognosis. The fact has been alluded to under a previous head, that it shows change in the course of disease before other symptoms can. Individual diseases have their peculiarities of thermometric range, and a marked variation from this indicates a grave or favorable termination. In typhoid fever an evening temperature of 103.5° shows a mild case; if it rises to 105°, dropping only to 104° in the morning, it is very grave. In pneumonia, 104° shows a severe attack, especially coming on early in its course. All diseases are not alike in regard to the height to which the thermometer may go within the bounds of safety. Cases of sunstroke have been recovered from in which the temperature of 110° has been reached; and Dr. Wilson Fox reports, in the London *Lancet*, the same height, in a case of rheumatism, which recovered. A few weeks ago I took the temperature for several days in a case of pneumonia, and finally found a morning temperature of 104.2°, pulse 112, and respiration 32. The evening temperature of the same day was 102.8°, which, being so decided a fall from morning to evening, was evidence that the crisis was past, although the pulse was accelerated to 120, and the respiration to 48. The fall here was not sufficient for the most practiced hand to detect. In a case of typhoid, looked upon as grave by the attending physician, I was enabled by the thermometer to give a favorable prognosis.

Can a diagnosis be made by the daily fluctuations of the thermometer? It would not be believed at first thought that any one would ever be driven to this means of diagnosis, but I have seen a case of gummy tumor of the brain, which in good hands was thought to be typhus fever, and this instrument would settle the question between them with certainty. Some diseases running a regular course have a typical range of temperature, so that one familiar with the subject, turning over the records of vital signs, can distinguish them one from another. In typhoid fever we see a good deal of regularity of the temperature line. Mounting gradually during the first week up above 100°, it holds there, with slight morning fall, till about the twenty-third day, when a considerable fall takes place, and we see the peculiarity of a nearly normal morning temperature and a high evening exacerbation, making long lines in the fever-curve. Typhus, again, is like typhoid, only the morning fall comes one week earlier. In the eruptive fevers generally the evening exacerbation is slight, the fever line, instead of being zigzag, running in a general straight line upward until a certain stage of the disease, and then downward in a likewise straight line. An intermittent of quotidian type shows a fever-line quite similar to that of the third week of typhoid; the tertian differs only in the skipping of one day. These are specimens of the typical thermometric ranges of diseases. It is evident how often diagnosis, that difficult branch of medicine, may be helped out by the thermometer.

Thermometry gives indications for treatment. An exceedingly high temperature is of itself dangerous to life from its effect on the nerve-centres, and should be treated by active antiphlogistics, regardless of the primary disease. This is illustrated by the cases reported by Wilson Fox, of rheumatism, in which the temperature rose to 110°, and which recovered under the use of cold douches and baths. A very forcible illustration of the value of the thermometer, as a help in treatment, is seen in pericarditis occurring in the course of rheumatism. Every case of this disease should be watched carefully with the thermometer, for by it we get premonitions of the occurrence of pericarditis, and are enabled to get the start of it. We are told, and very properly, to listen every day to the heart-sounds, but the fact is evident that, if you wait for cardiac murmurs, you are waiting simply till the serous surfaces are covered with exudation to produce them. I have seen one case in which by most careful examination no murmurs were heard, but the autopsy showed four ounces of bloody serum in the pericardium, and the surfaces covered with a fibrinous exudate presenting the "bread-and-butter" appearance. The serum separated the membranes and prevented friction-sounds. The pulse is sensitive to acute disease of the heart, but it is liable to extraneous disturbance. So, too, with the respiration. And, more than all, thermometric change occurs before anything else, and so is premonitory, in this and other diseases.

Febrile action is inconsistent with health, and an instrument which indicates and measures it, translates what is going on in the system. The fever is in many cases a safer guide than anything else. The pulse is disturbed by nervous excitement; exertion increases the respiration; the countenance gives valuable indications in sick children especially, but often it tells nothing; the history cannot always be gained clearly. But the temperature can always be taken, and has one advantage over most other means of diagnosis: it is uninfluenced by extraneous circumstances, and always tells the truth. Its language is to be learned by study and observation.

To sum up in a sentence the valuable points of the thermometer: it shows the existence of febrile exacerbation; it is premonitory of change in the course of a disease; it helps in diagnosis, prognosis, and treatment; and its indications are uninfluenced by any outside circumstance whatsoever.—*N. Y. Med. Jour.*

